# Exploring the utility of organo-polyoxometalate hybrids to inhibit SOX transcription factors

**DOI:** 10.1186/2045-9769-3-10

**Published:** 2014-07-19

**Authors:** Kamesh Narasimhan, Kevin Micoine, Emmanuel Lacôte, Serge Thorimbert, Edwin Cheung, Bernold Hasenknopf, Ralf Jauch

**Affiliations:** 1 Donnelly Centre for Cellular and Biomolecular Research, University of Toronto, Toronto M5S 3E1, Canada; 2 Genome Institute of Singapore, 60 Biopolis Street, Buona Vista 138672, Singapore; 3 Sorbonne Universités, UPMC Univ Paris 06, Institut Parisien de Chimie Moléculaire, UMR 8232, 4 Place Jussieu, 75005 Paris, France; 4 CNRS, Institut Parisien de Chimie Moléculaire, UMR 8232, 4 Place Jussieu, Paris, 75005, France; 5 Faculty of Health Sciences, University of Macau, Av. Padre Tomas Pereira, Taipa, Macau, China; 6 Genome Regulation Laboratory, Drug Development Pipeline, Guangzhou Institutes of Biomedicine, 190 Kai Yuan Avenue, Science Park, 510530 Guangzhou, China

## Abstract

**Background:**

SOX transcription factors constitute an attractive target class for intervention with small molecules as they play a prominent role in the field of regenerative biomedicine and cancer biology. However, rationally engineering specific inhibitors that interfere with transcription factor DNA interfaces continues to be a monumental challenge in the field of transcription factor chemical biology. Polyoxometalates (POMs) are inorganic compounds that were previously shown to target the high-mobility group (HMG) of SOX proteins at nanomolar concentrations. In continuation of this work, we carried out an assessment of the selectivity of a panel of newly synthesized organo-polyoxometalate hybrids in targeting different transcription factor families to enable the usage of polyoxometalates as specific SOX transcription factor drugs.

**Results:**

The residual DNA-binding activities of 15 different transcription factors were measured after treatment with a panel of diverse polyoxometalates. Polyoxometalates belonging to the Dawson structural class were found to be more potent inhibitors than the Keggin class. Further, organically modified Dawson polyoxometalates were found to be the most potent in inhibiting transcription factor DNA binding activity. The size of the polyoxometalates and its derivitization were found to be the key determinants of their potency.

**Conclusion:**

Polyoxometalates are highly potent, nanomolar range inhibitors of the DNA binding activity of the Sox-HMG family. However, binding assays involving a limited subset of structurally diverse polyoxometalates revealed a low selectivity profile against different transcription factor families. Further progress in achieving selectivity and deciphering structure-activity relationship of POMs require the identification of POM binding sites on transcription factors using elaborate approaches like X-ray crystallography and multidimensional NMR. In summary, our report reaffirms that transcription factors are challenging molecular architectures and that future polyoxometalate chemistry must consider further modification strategies, to address the substantial challenges involved in achieving target selectivity.

## Background

**Figure 1:**
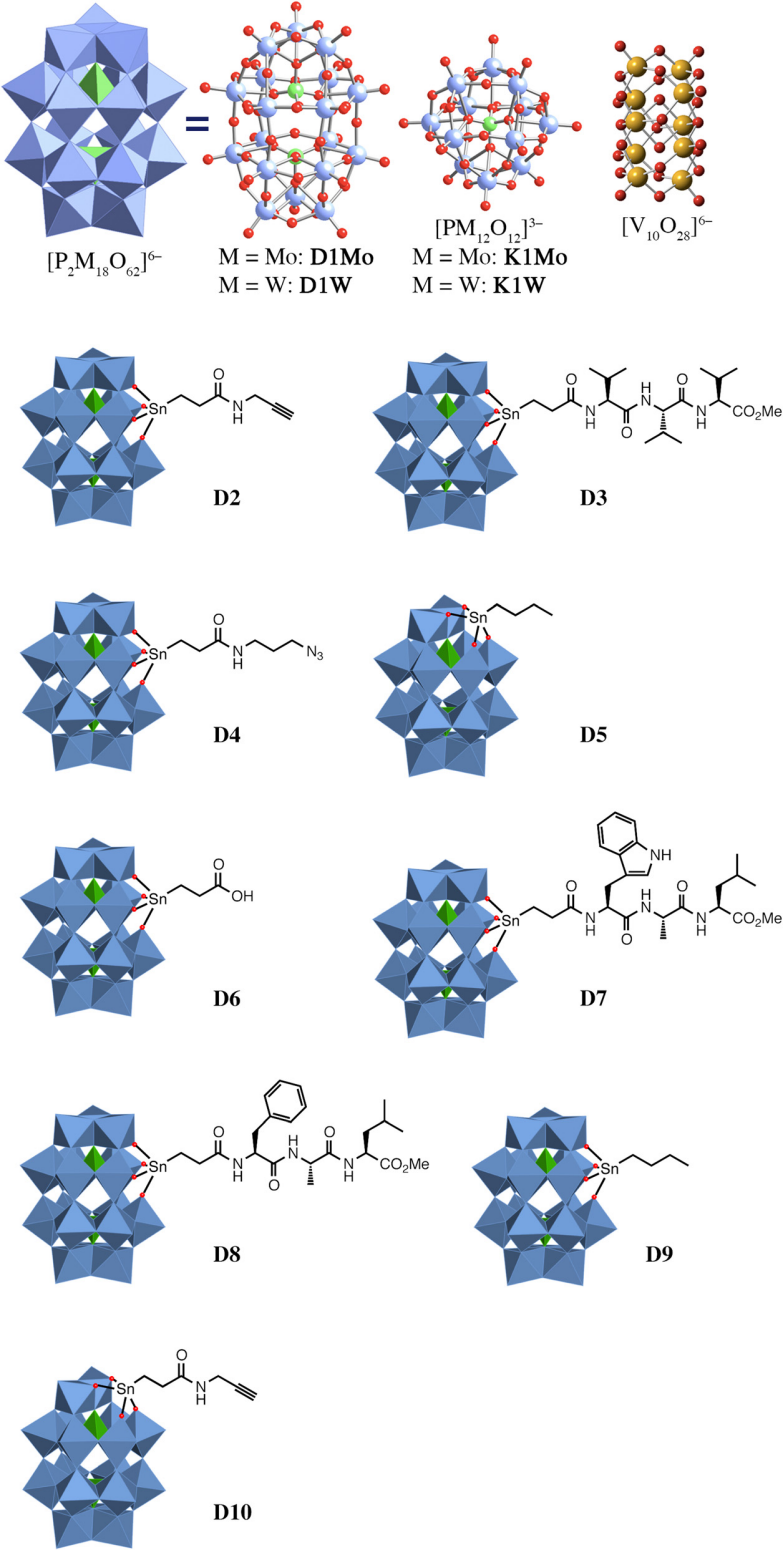
**The panel of polyoxometalates used in this study.** Compound acronyms and the chemical formulas are as provided in Table 1.

Transcription factors (TFs) with critical functions in cancer and stem-cell biology are desirable targets for small molecule inhibition [1,2]. In particular, members of the SOX TF family were reported to drive cancer progression [3,4]. However, chemical inhibitors of SOX proteins that would have great potential to counteract oncogenesis are presently not available. Some of the best selling drugs approved by the FDA (Food and drug administration) are in fact known to target TFs [5]. However, those drugs do not bind the DNA binding domains (DBDs) of TFs because of their highly electrostatic nature, the lack of binding pockets, and the structural dynamics of TFs in the absence of DNA [6]. We hypothesized that the negatively charged Polyoxometalates (POMs) provide a suitable scaffold for targeting DBDs [7]. POMs are nanometer sized inorganic oxyanions comprising transition metals belonging to Group 5 and 6 of the periodic table in their highest oxidation states [8]. The metals are held together by oxygen atoms and often enclose one or more central heteroatoms like phosphorus or silicon. Some common structural POM families of importance in the field of biomedicine are the Keggin [XM_12_O_40_]^n-^, and the Dawson structure [X_2_M_18_O_62_]^n–^ where M is the transition metal atom (typically tungsten or molybdenum), X is the heteroatom (typically phosphorous) and n is the number of ionic charges (Figure 1) [8].

A variety of biological effects of POMs are documented [9-15], including antitumor activity [16-19]. More recently, the effective inhibition of various unrelated enzymes has also been reported [20-25]. We previously identified the Dawson phosphomolybdate (D1Mo: K_6_ [P_2_Mo_18_O_62_]) as a nanomolar inhibitor of the Sox2-HMG domain [7]. Although this Dawson-POM was found to be a rather potent inhibitor of SOX-DNA interaction, it exhibits only moderate selectivity. To optimize selectivity we now build upon this previous study and examined the potential of a larger panel of POMs, including novel organo-hybrids, and an expanded set of TFs.

## Materials and methods

To assess the selectivity of a panel of POMs, residual DNA binding activity experiments were carried out using different members of the Sox family and structurally unrelated TFs such as Pax6, REST, FoxA1 and AP-2γ. The mouse REST Cys2His2 zinc finger protein and the HMG domains of the Sox paralogs Sox4, 5, 6, 7, 8, 9, 10, 11, 17 and 18 were purified using previously published protocols [26,27]. Full length human AP-2γ and full length FoxA1 proteins were prepared as described [28,29]. Prior to carrying out selectivity assays, a 20 μM working stock of the polyoxometalates was created in a 100% DMSO solution. The buffer solution for the residual DNA binding experiments had the final working composition of 10 mM Tris pH 8.0 and 100 mM KCl prepared with molecular grade water. The final DMSO composition in the binding buffer was maintained at 2% v/v in the selectivity assays. Hence addition of 2% DMSO alone acts as a negative control for the assay, as DMSO at 2% v/v does not influence the TF-DNA complex and hence the residual DNA binding activity measurements. Sox2-HMG domain was previously shown to be inhibited by the Dawson POM D1Mo (K_6_ [P_2_Mo_18_O_62_]) at an IC_50_ of 98.6 ± 22.1 nM [7]. Using the IC_50_ of Sox2 inhibition by the unmodified Dawson POM D1Mo (K_6_ [P_2_Mo_18_O_62_]) as a reference, all the different inhibitor compounds were added at a concentration of 125 nM to 15 different preassembled TF-DNA complexes. TF concentrations were chosen such that ~ 70-90% of the FAM-labeled DNA was bound. The residual DNA binding activities of TFs were then obtained either by fluorescence anisotropy measurements or by quantifying EMSAs (Electrophoretic Mobility Shift assays). The residual DNA binding activity is calculated with reference to the fraction of the maximally bound TF-DNA complex in the presence of 2% DMSO alone (without compound treatment) and is reported as a percentage of the fraction of bound TF-DNA complex before and after compound addition. Hence 0% residual binding activity would correspond to maximum inhibition, while 100% activity would correspond to no inhibition. For Pax6, residual DNA binding activity was measured by EMSA (Additional file 1: Figure S1) under the same condition as the fluorescence anisotropy measurements, as Pax6 did not exhibit a significant change in anisotropy values upon complex formation. The final reported residual DNA binding activity is an average of five independent experiments.

The FAM labeled DNA elements used in the binding experiments were *HPSE:*5’(FAM)-AAAGTGCCCAGAGCCCATG-3’;*RE-1:*5’(FAM)-CTTCAGCACCTCGGACAGCTCC-3’; *FoxA1:*5’(FAM)-TGCCAAGTAAATAGTGCAG-3’;*Pax*:5’(FAM)-AAGCATTTTCACGCATGAGTGCACAG-3’;*CCND1:*5’(FAM)-CTGCCGGGCTTTGATCTTTGCT-3’, where *RE1, HPSE*, *FoxA1, Pax* and *CCND1* elements contain cognate binding sites of REST, AP-2γ, FoxA1, Pax6 and Sox-HMG proteins respectively. Labeled reverse complementary strands of the above sequences were used to generate DNA duplexes.

Sodium phosphomolybdate Na_3_ [PMo_12_O_40_], Sodium phosphotungstate Na_3_ [PW_12_O_40_] and Sodium metatungstate Na_6_W_12_O_39_ · xH_2_O were purchased from Sigma-Aldrich. The general synthesis and ligation procedures of organic substrates to Dawson POMs are outlined elsewhere [30,31]. Bulk amounts of Dawson phosphomolybdate K_6_ [P_2_Mo_18_O_62_] were also obtained by custom synthesis from Asischem Inc.

## Results and discussions

**Figure 2:**
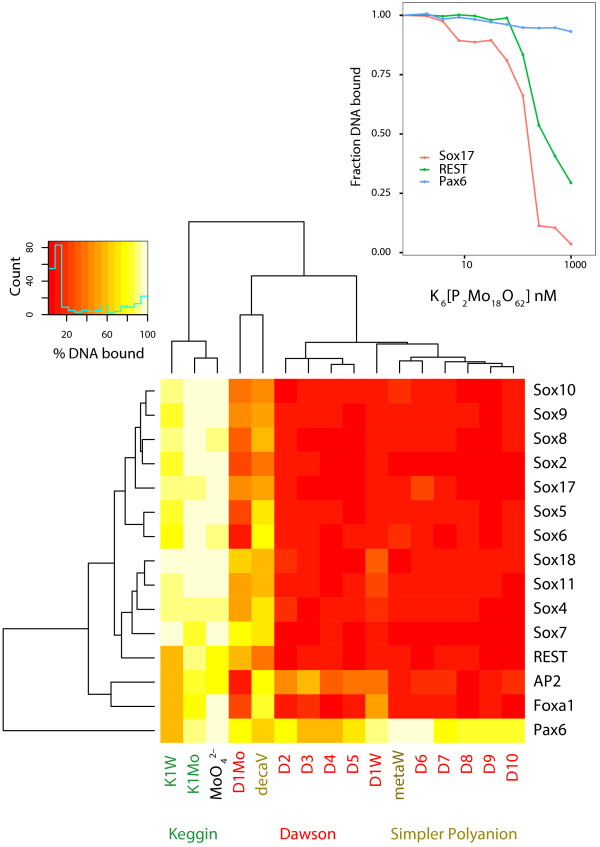
**A heatmap displaying the average residual DNA binding activities of 15 different TFs upon treatment with 125 nM of a panel of inhibitor compounds.** The residual DNA binding activity is reported as an average of five independent experiments. Two-dimensional clustering of the residual DNA binding activities was carried out by hierarchical clustering analysis (Euclidean distance). (Red color indicates high inhibition, while yellow color indicates relatively lesser inhibition). Inhibitor compounds are color coded by their POM class. Inset shows typical inhibition profiles of representative TFs namely Sox17, REST and Pax6 upon treatment with Dawson POM K_6_ [P_2_Mo_18_O_62_] (D1Mo), measured by fluorescence anisotropy.

In total, the residual DNA binding activities of 15 different TFs were estimated against a panel of inhibitors that could be broadly classified into “Keggin”, “Dawson” and “simpler polyanion” (metatungstate, sodium molybdate and decavanadate) types. The structures, compound acronyms and the chemical formulas of POMs employed in this study are given in Figure 1 and Table 1. The mean residual DNA binding activity of TFs from five independent experiments is displayed as a heatmap after hierarchical clustering analysis using the “R heatmap.2” package (Figure 2) [32].

**Table 1:** Panel of inhibitor compounds screened for inhibition of DNA binding activity of 15 transcription factors

**POMs**	**Chemical formula**	**Type**	**Organic side chain**	**Mol.wt**
**D1Mo**	K_6_ [P_2_Mo_18_O_62_]	Dawson	None	3016
**D1W**	K_6_ [P_2_W_18_O_62_]	Dawson	None	4597
**D2**	(NC_16_H_36_)_7_ α_1_-[P_2_W_17_O_62_SnC_6_H_8_N_4_]	Dawson	Aliphatic	6089
**D3**	(NC_16_H_36_)_7_ α_2_-[P_2_W_17_O_62_SnC_16_H_23_N_3_O_2_]	Dawson	Val-Val-Val	6357
**D4**	(NC_16_H_36_)_7_ α_2_-[P_2_W_17_O_62_SnC_6_H_11_N_4_O]	Dawson	Aliphatic	6135
**D5**	(NC_16_H_36_)_6_ α_2_-[P_2_W_17_O_61_SnC_4_H_10_]	Dawson	Aliphatic	5794
**D6**	(NC_16_H_36_)_6_ α_2_-[P_2_W_17_O_62_SnC_3_H_4_O_2_]	Dawson	Aliphatic	5810
**D7**	(NC_16_H_36_)_7_ α_1_-[P_2_W_17_O_66_SnC_24_H_34_N_4_]	Dawson	Trp-Ala-Leu	6438
**D8**	(NC_16_H_36_)_7_ α_1_-[P_2_W_17_O_66_SnC_22_H_32_]	Dawson	Phe-Ala-Leu	6356
**D9**	(NC_16_H_36_)_6_ α_1_-[P_2_W_17_O_61_SnC_4_H_10_]	Dawson	Aliphatic	5794
**D10**	(NC_16_H_36_)_7_ α_2_-[P_2_W_17_O_62_SnC_6_H_8_N_4_]	Dawson	Aliphatic	6089
**K1Mo**	Na_3_ [PMo_12_O_40_]	Keggin	None	1892
**K1W**	Na_3_ [PW_12_O_40_]	Keggin	None	2946
**decaV**	(NC_16_H_36_)_3_ [H_3_V_10_O_28_]	Decavandate	None	1688
**metaW**	Na_6_W_12_O_39_ · xH_2_O	Sodium metatungstate	None	2969
**MoO**_**4**_^**2−**^	Na_2_MoO_4_ · 2H_2_O	Sodium Molybdate	None	206

The Dawson POMs were found to be highly potent in their inhibition profiles of not only the Sox-HMG members but also other TF families like FoxA1, REST and AP2 (Additional file 2: Table S1). The simpler polyanion, sodium molybdate serves the purpose of being an external negative control in our assay and does not cause inhibition of any TF DNA complex. Overall, among the TFs tested it was observed that Pax6 was the most inert to treatment with POMs. The only POMs that were relatively effective in inhibiting Pax6 activity were D3, D4 and K1W (Figure 2). From the clustering analysis it could be observed that the Keggin and Dawson POMs exhibit a marked dichotomy in their selectivity and inhibition potential of TFs. Keggin POMs exhibit relatively lower inhibition potency as compared to the Dawson POMs on all TF families studied, suggesting that the size or charge of POMs is an important consideration in the inhibition of TFs (Figure 2). Keggin ions are almost spherical and about 1 nm in diameter with 3– charge, whereas Dawson ions are close to an elongated cylinder of about 1.5 × 1 nm with 6– or 7– charge. Metatungstate has the size and shape of the Keggin ion, but a 6– charge similar to the Dawson ion. Its high inhibition effect in the same range as the Dawson POMs tends to privilege charge as the discriminating factor for inhibition. The organically modified Dawson POMs slightly amplified the inhibitory potency of the unmodified Dawson scaffold D1W (K_6_ [P_2_W_18_O_62_]). The side chain increases the steric bulk and the substitution of a {WO}^4+^ by a {SnR}^3+^ fragment also increases the negative charge of the framework. Taken together, we surmise that a good POM inhibitor must be at least 1 nm in diameter and should carry a charge of 6– or more. This is consistent with previous observations, but a larger panel of POMs must be screened before a definite conclusion can be reached [10]. In any case, some caution should remain for structure-activity relationships because POMs undergo hydrolysis and condensation reactions in water, and the proteins might influence these equilibria. We believe that this phenomenon is responsible for the behavior of the Dawson molybdate (D1Mo: K_6_ [P_2_Mo_18_O_62_]) and the decavanadate (decaV). These compounds are known to be hydrolytically very unstable at neutral pH and inhibition is only observed with a protein-dependent stabilization of the active form [23].

Modified organic Dawson POMs were tested under the assumption that their organic side-chains would be capable of enabling selective inhibition, however the outcome of testing a limited sub-set of modified Dawson POMs indicates substantial challenges involved in achieving selectivity against different TF DNA binding domains. The modified Dawson POMs showed amplified potency without exhibiting selectivity between various TFs. The amplified potency of the modified Dawson POMs when compared to the unmodified Dawson POM is presumably due to size and charge effects as discussed above. Interactions of the organic side chains with TFs might furthermore occur, but are not sufficiently discriminating here. Further progress in this regard, requires the identification of the Dawson POM binding sites on the Sox-HMG domain by higher resolution techniques like X-ray crystallography and multi-dimensional NMR, before optimally designed organic side chains can be grafted onto the inorganic scaffold.

Even though targeting the DNA binding domains of TFs is challenging, evolving a core inhibitor scaffold that can be functionally customized to achieve specificity will have a significant impact in developing drugs that target TFs. Recent advancements in click chemistry to incorporate a range of organic substrates in tin substituted Dawson POMs has expanded the horizon of organic modifications that could be achieved in POM chemistry [30,31]. We propose that the potent POM based inhibition of TFs provides a powerful strategy and that target selectivity could be achieved by conjugation with natural biological molecules like carbohydrates, steroids and peptides. It can be envisaged that in the future, subsets of TF interaction domains or even whole peptides conjugated with synthetic Dawson POMs will spawn the development of a newer breed of POMs that can be eventually tested for targeting regulatory or DNA binding domains in a truly selective way.

The selectivity assay described thus far has been carried out *in vitro* using short DNA molecules and isolated DNA binding domains at a non-neutral pH (pH 8.0). Any conclusive assessment and future development of synthetic Dawson POMs also requires that these experiments be extended further onto cell-lines and animal models where a multitude of variables like protein co-factor interactions, salinity and pH will affect the final drug response. In this regard, it has been noted that POMs are large and highly negatively charged rendering their penetration of cellular membranes a serious challenge [33]. However, several advancements like encapsulation of POMs in liposomes have been achieved to overcome this challenge of delivery into cells [33]. Overall, the results shown here reaffirms that transcription factors are indeed challenging molecular architectures and that polyoxometalate platforms like the Dawson phosphotungstate which combine inhibition potency and easy chemical modification should be further systematically explored to truly achieve target selectivity.

## Abbreviations

POMs: Polyoxometalates; TFs: Transcription factors; DBD: DNA binding domain; EMSA: Electrophoretic Mobility Shift assay.

## Competing interests

The authors declare that they have no competing interests.

## Authors’ contributions

KN carried out the work and has written the first draft of the paper. KM, BH, EL and ST carried out the chemical synthesis. EC provided critical reagents. KN, BH and RJ conceived the study, participated in the analysis and coordination and helped drafting of the final manuscript. All authors read and approved the final manuscript.

## Supplementary Material

Additional file 2: Table S1 Residual DNA binding activities (in %) of 15 TFs upon treatment with a panel of inhibitors. The residual DNA binding activity is expressed as mean ± relative standard deviation from five independent experiments. Click here for file

Additional file 1: Figure S1 The inhibition of Pax6 by a panel of inhibitor compounds studied using EMSA. Residual DNA binding activity was estimated from maximally bound Pax6-DNA (no POM, 2% DMSO) and free DNA gel-shift intensities (Pax6 DNA alone) as reference. Click here for file
