# Supplementary material for: Exploring the utility of organo-polyoxometalate hybrids to inhibit SOX transcription factors
Source: Cell Regen. 2014 Jul 19;3:10. doi: 10.1186/2045-9769-3-10 (PMC4306199; doi:10.1186/2045-9769-3-10)
Supplement: Additional file 1: Figure S1 — The inhibition of Pax6 by a panel of inhibitor compounds studied using EMSA. Residual DNA binding activity was estimated from maximally bound Pax6-DNA (no POM, 2% DMSO) and free DNA gel-shift intensities (Pax6 DNA alone) as reference. [file 2045-9769-3-10-S1.pdf]

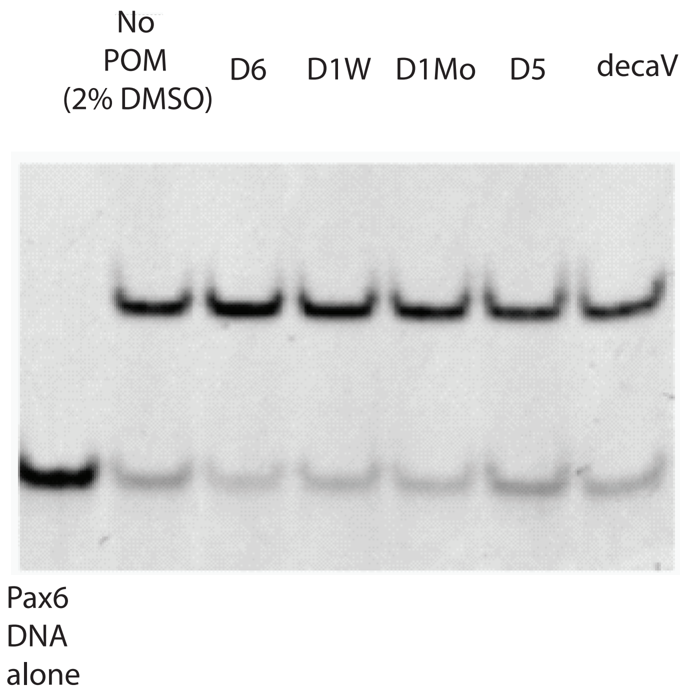
No POM (2% DMSO)
D6
D1W
D1Mo
D5
decaV
Pax6 DNA alone

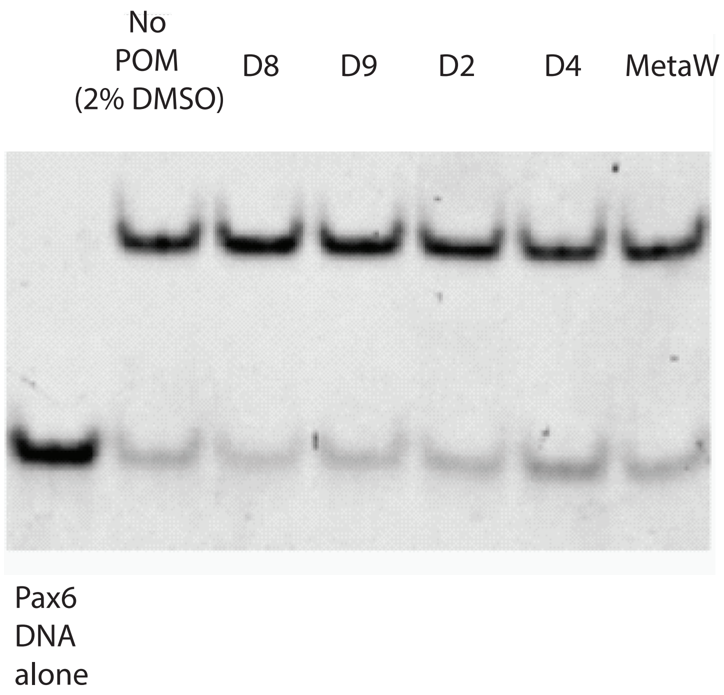
No POM (2% DMSO)
D8
D9
D2
D4
MetaW
Pax6 DNA alone

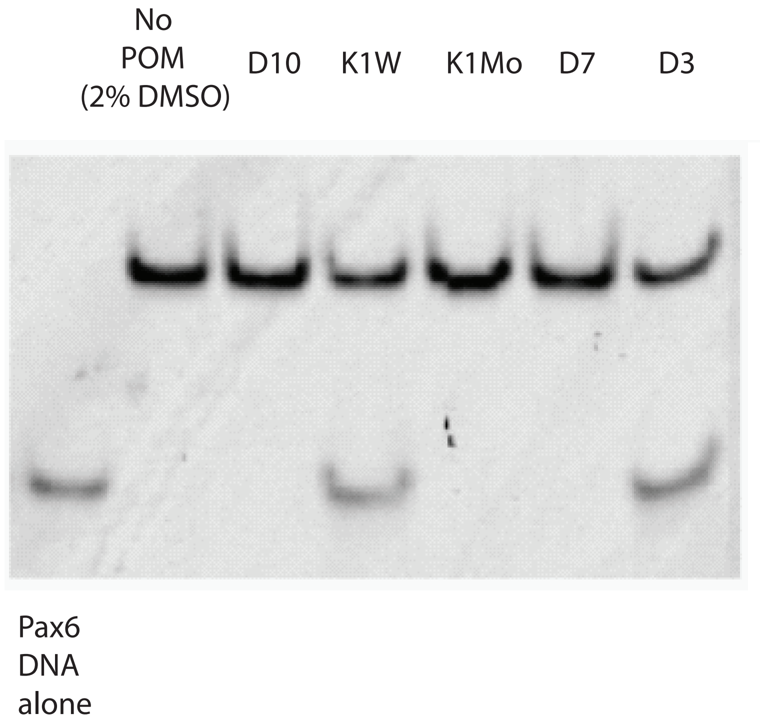
No POM (2% DMSO)
D10
K1W
K1Mo
D7
D3
Pax6 DNA alone
